# Discovering trends and hotspots of biosafety and biosecurity research via machine learning

**DOI:** 10.1093/bib/bbac194

**Published:** 2022-05-22

**Authors:** Renchu Guan, Haoyu Pang, Yanchun Liang, Zhongjun Shao, Xin Gao, Dong Xu, Xiaoyue Feng

**Affiliations:** 1 Key Laboratory of Symbolic Computation and Knowledge Engineering of the Ministry of Education, College of Computer Science and Technology, Jilin University, Changchun, 130012, Jilin, China; 2 Zhuhai Sub Laboratory, Key Laboratory of Symbolic Computation and Knowledge Engineering of the Ministry of Education, Zhuhai College of Science and Technology, Zhuhai, 519041, Guangdong, China; 3 Department of Epidemiology, Ministry of Education Key Laboratory of Hazard Assessment and Control in Special Operational Environment, School of Public Health, Air Force Medical University, Xi’an, 710032, Shaanxi, China; 4 Computational Bioscience Research Center, King Abdullah University of Science and Technology (KAUST), Thuwal, 23955, Saudi Arabia; 5 Computer, Electrical and Mathematical Sciences and Engineering Division, King Abdullah University of Science and Technology (KAUST), Thuwal, 23955, Saudi Arabia; 6 BioMap, Beijing, 100192, China; 7 Department of Electric Engineering and Computer Science, and Christopher S. Bond Life Sciences Center, University of Missouri, Columbia, 65201, Missouri, USA

**Keywords:** biosafety and biosecurity, COVID-19, information retrieval, machine learning

## Abstract

Coronavirus disease 2019 (COVID-19) has infected hundreds of millions of people and killed millions of them. As an RNA virus, COVID-19 is more susceptible to variation than other viruses. Many problems involved in this epidemic have made biosafety and biosecurity (hereafter collectively referred to as ‘biosafety’) a popular and timely topic globally. Biosafety research covers a broad and diverse range of topics, and it is important to quickly identify hotspots and trends in biosafety research through big data analysis. However, the data-driven literature on biosafety research discovery is quite scant. We developed a novel topic model based on **l**atent **D**irichlet **a**llocation, **a**ffinity **p**ropagation clustering and the **P**age**R**ank algorithm (LDAPR) to extract knowledge from biosafety research publications from 2011 to 2020. Then, we conducted hotspot and trend analysis with LDAPR and carried out further studies, including annual hot topic extraction, a 10-year keyword evolution trend analysis, topic map construction, hot region discovery and fine-grained correlation analysis of interdisciplinary research topic trends. These analyses revealed valuable information that can guide epidemic prevention work: (1) the research enthusiasm over a certain infectious disease not only is related to its epidemic characteristics but also is affected by the progress of research on other diseases, and (2) infectious diseases are not only strongly related to their corresponding microorganisms but also potentially related to other specific microorganisms. The detailed experimental results and our code are available at https://github.com/KEAML-JLU/Biosafety-analysis.

## Introduction

**Figure 1:**
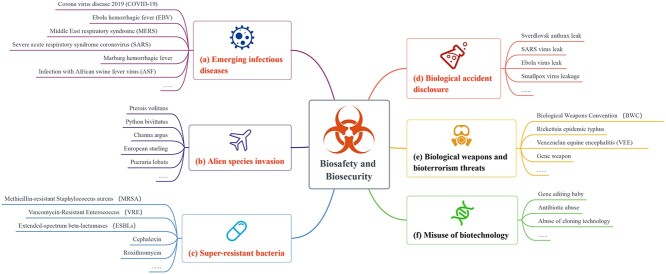
The subdivisions of biosafety research include ‘alien species invasion’, ‘emerging infectious diseases’, ‘superresistant bacteria’, ‘misuse of biotechnology’, ‘biological weapons and bioterrorism threats’ and ‘biological accident disclosure’. It is worth mentioning that medical papers do not fully cover all areas of biosafety research. For some studies related to politics, military, ethics, sociology and other disciplines, our data have little or no relevance to them. For some medicine-related biosafety research, there is considerable overlap between different segments. We believe it is necessary to present these results based on the experimental data in a more scientific and intuitive way. And the topics involved in the paper are colored orange.

‘Biosafety’ refers to safety issues caused by infectious diseases, alien species invasion, biological weapons, biotechnology abuse, loss of biological resources and laboratory safety accidents [74]. Related research has been conducted in medicine [3], biology [44], chemistry [49], environmental science [13] and other disciplines, covering medicine and health, agriculture, the military, science and technology, education, the environment and areas such as monitoring, forecasting, detection, tracing, prevention and control, diagnosis, treatment and other key technical fields [66]. The main content related to biosafety is shown in Figure 1.

With the development of biotechnology and the advancement of globalization [18, 30], the notion of biosafety has gradually become better defined. The international community’s attention to biosafety is increasing, and prevention and emergency systems have been rapidly established [10, 21, 63]. Since the proposal of the *2000 Cartagena Protocol of Biosafety* [19], many countries have adopted this protocol. Unfortunately, outbreaks of major infectious diseases [42] and biosafety incidents [69] have also brought major challenges to biosafety-related work.

As an important emerging aspect of global security, biosafety challenges come from many areas. Large-scale outbreaks of **infectious diseases** (Figure 1a) are the most crucial challenge. In the past 10 years alone, more than a dozen major international infectious disease incidents have occurred. In 2013, the H1N1 swine flu virus broke out. There were a final total of 12 033 laboratory confirmed cases, including 805 deaths [61]. The Ebola virus was first discovered in Central Africa in 1976. In 2014, an Ebola epidemic broke out again and rapidly spread almost completely out of control. The associated mortality rate was as high as 40.3%, causing a global panic. At present, it is still a priority disease of the WHO. It was not until November 2019 that the Ebola hemorrhagic fever vaccine was approved for the first time [62]. As of July 2015, no medication had been proven safe and effective for Ebola treatment. By August 2019, only two experimental treatments had been found to be 90% effective in treating this infectious disease [57].

Similarly, **human-induced biosafety accidents** (Figure 1d) are a major challenge for biosafety [2]. Laboratory infections threaten the health of workers and may even cause accidental leakage of organisms, which can have a major impact on the global public health system [23, 25]. During the 7-year span of 2004 to 2010, the US Centers for Disease Control and Prevention reported 727 biological media loss and leakage incidents, of which 639 were leakage incidents and most of them Biosafety Level 3 (BSL-3) incidents [69].

In addition, the international community faces **multidrug-resistant bacteria** (Figure 1c), a common biosafety problem [64]. Antimicrobial resistance (AMR) has had a significant impact worldwide. A working report published by the UK government in December 2014 predicted that drug-resistant bacteria would reduce the global gross domestic product (GDP) by 2–3.5% by 2050 [74]. In 2019, approximately 230 000 people died from infections featuring drug-resistant bacteria strains in the United States, and the related treatment cost the US medical system more than $200 trillion [74]. Since some biosafety-related fields have little to do with biological research, we do not have a great deal of information on them.

The biosafety challenges facing the international community also include biological weapons (Figure 1e) [59], misuses of biotechnology (Figure 1f) and biological accidents (Figure 1d) [1], invasions of alien species (Figure 1b) [54] and bioterrorism (Figure 1e) [14]. To solve these problems, many researchers have begun to engage in biosafety research.

With the development of biosafety research, related papers and data have been produced at a speedy rate. In PubMed (https://pubmed.ncbi.nlm.nih.gov/), 66 758 biosafety-related papers have been published in the last 10 years. For researchers, it has become a great challenge to quickly and effectively identify the latest research progress and results from among the tens of thousands of publications. Additionally, extracting the research hotspots and predicting trends are difficult problems.

**Figure 2:**
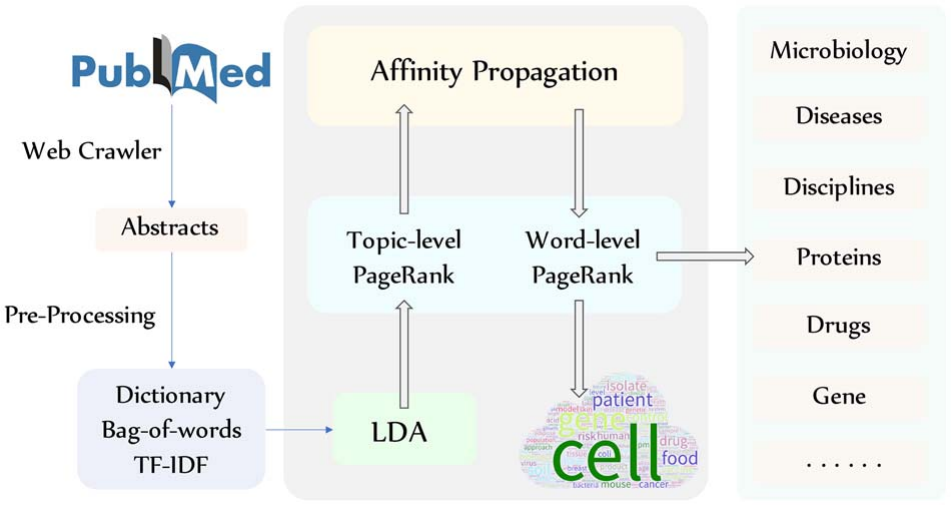
Overall framework and workflow of the LDAPR model. The data were downloaded from the PubMed database and preprocessed in Python. The preliminary results were obtained through a model composed of LDA, AP and a hierarchical weighted PageRank algorithm. The results could then be used for multiple data analysis purposes such as retrieval, sorting and visualization.

In response to these issues, we collected the abstracts of biosafety-related papers released in the past 10 years from PubMed and designed a novel topic model called LDAPR (**l**atent **D**irichlet **a**llocation (LDA), the **a**ffinity **p**ropagation (AP) algorithm and the hierarchical **P**age**R**ank algorithm) to extract the topics of these abstracts. Figure 2 shows the overall framework and workflow of LDAPR. This framework greatly reduces the dependence on human knowledge and labor and produces accurate and high-quality results. In addition, by introducing medical subject heading (MeSH) term categories, we carried out classification and trend analysis, focusing on the categories of microorganisms, diseases, drugs, disciplines and regions, and we further explored the relationships between them.

## Results

### Overview

In the following sections, we discuss the results from the following seven viewpoints: topic results, microorganism keywords, disease keywords, disciplinary keywords, regional keywords, research trends and challenges and topics over the decade.

### Topic results

First, we visualize the annual results with word clouds. Every word cloud represents a topic and contains 30 words. The higher the weight is, the larger the word. If we take the word clouds of 2020 as an example, as shown in Figure 5, there are 10 word clouds in total, and their centers are ‘virus’, ‘food’, ‘plastic’, ‘pneumococcal’, ‘tolerance’, ‘utilization’, ‘flavonoid’, ‘cell’ and ‘epoxide’. Most words under the same topic are related. For example, the central word in the first word cloud is ‘virus’, and the secondary central words are ‘infectious’, ‘patient’, ‘ZIKV [Zika virus]’ and other virus-related words. As the culprit behind many diseases, viruses cause many serious biosafety problems [58]. Research on viruses is an important aspect of biosafety work. In addition, another central word, ‘food’, is a research hotspot in biosafety. Its representative topics include food safety-related words such as ‘beef’, ‘milk’ and ‘chocolate’. Most historical biosafety issues are closely related to food safety [46].

Table 1 shows the central topic words over the years. Among them, ‘cell’, ‘influenza’, ‘risk’ and other words related to biosafety appear many times, while some words, such as ‘island’, appear only in a particular year (2017); we found relevant research hotspots on biosecurity and islands published in that year [47, 70].

**Table 1:** Central words in each year; the words strongly related to biosafety are in **bold**.

Year	Central word
2011	waste, bovine, device, vessel, **lactobacillus**, **anti-inflammatory**, **cereus**, juice, **virus**, community
2012	**transfusion**, **antibacterial**, **salmonella**, **pharmacokinetics**, subject, enterica, cell, facility, pig, combination, **listeria**
2013	**transgenic**, milk, chemical, cell, **monocytogenes**, food, health, **influenza**, sausage, climate, evolutionary, severe, drink
2014	ultrasound, plasma, egg, internalization, **spore**, **virus**, **vaccine**, **mycotoxin**, ovarian, **salmonella**, biofilm
2015	**virus**, rat, copper, embryo, soil, waste, hepatocellular, chemical, irrigation, metastatic, medicine, infant, inhibitor
2016	freshwater, cell, health, oil, milk, **strain**, cereus, prostate, diet, cartilage, worker, cell, multiplex, graphene
2017	**infection**, adipose, isolates, cell, metastasis, grain, artery, **arsenic**, food, **virus**, bat, child, **pneumococcal**
2018	water, reproductive, embryonic, therapy, horse, cell, assembly, **pneumoniae**, dental, milk, diet, **virus**, soil
2019	**zikv**, **additive**, oxidative, **bacillus**, exosomes, apple, **aeruginosa**, pig, **hiv**, soil, chemical, farmer, **crohn**
2020	virus, **campylobacter**, food, epoxide, **melanoma**, utilization, **pneumococcal**, plastic, **flavonoid**, **tolerance**

By analyzing data from the past 10 years, we obtained 122 clusters, each containing 30 words. After deduplication, 1788 unique words were obtained, and then we used MeSH terms to identify and classify the results. A total of 32 different categories were found. We conducted in-depth research on 12 of the main categories by analyzing the hotspots and trends. The next sections show analyses of the keywords by microorganism, disease, discipline and region.

### Microorganism keywords

Keywords related to microorganisms appear most frequently in the results. We found references to 28 kinds of microorganisms in the data. They are ‘bacteriophage’, ‘severe acute respiratory syndrome coronavirus 2 (SARS-CoV-2/2019-nCoV/COVID-19)’, ‘Crimean-Congo hemorrhagic fever virus’, ‘Ebola virus’, ‘enterovirus’, ‘hepatitis A virus’, ‘human adenovirus’, ‘Middle East respiratory syndrome coronavirus (MERS-CoV)’, ‘rabbit hemorrhagic disease virus (RHDV)’, ‘Zika virus’, ‘aureus’, ‘Bacillus subtilis’, ‘Brucella’, ‘Campylobacter’, ‘Clostridium’, ‘cyanobacteria’, ‘Escherichia coli’, ‘Klebsiella’, ‘Lactobacillus’, ‘Lactococcus’, ‘Listeria monocytogenes’, ‘meningococcus’, ‘Salmonella’, ‘Serratia marcescens’, ‘Streptococcus pneumonia’, ‘Candida’, ‘Cryptococcus neoformans’ and ‘Pichia pastoris’. These can be divided into three categories: bacteria, viruses and fungi. For example, *Listeria monocytogenes*, Meningococcus and Salmonella are bacteria, and Zika virus is a virus.

**Figure 3:**
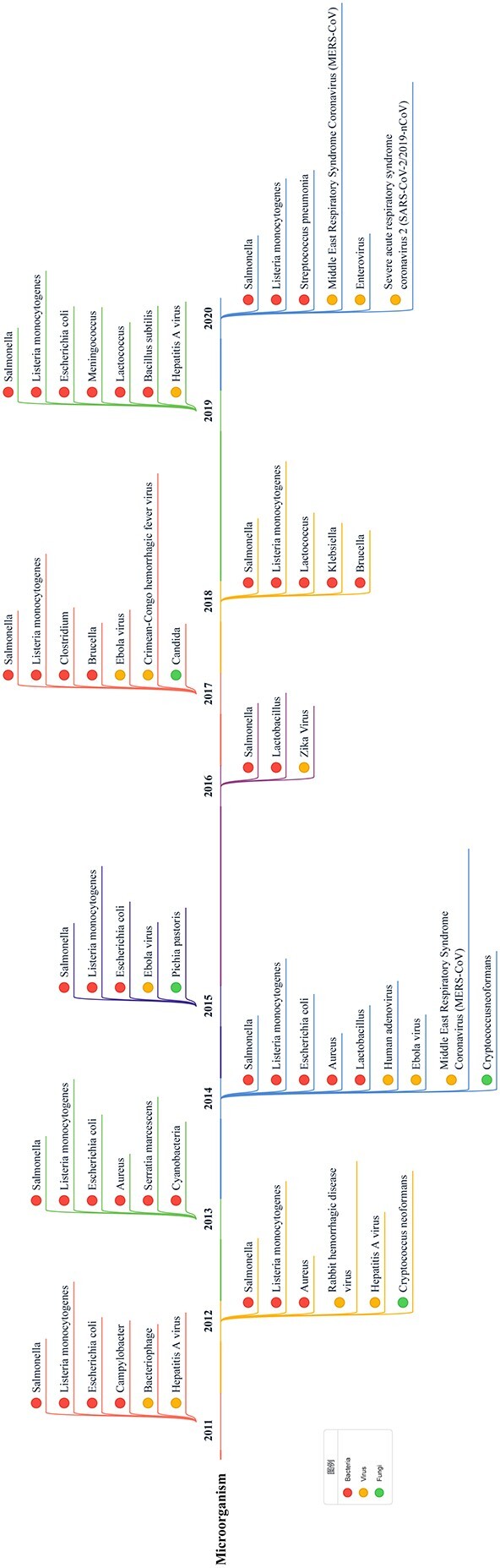
Microorganism-related keywords for each year from 2011 to 2020. We arrange the keywords in chronological order. Different-colored dots represent different types of microorganisms, including bacteria (red), viruses (yellow), and fungi (green). Since no other types of microorganisms (such as Mycoplasma) are found in the results, we do not establish an ‘other’ category.

Figure 3 shows the microorganism keywords by year (to make the descriptions more accurate, we mainly used the scientific names of the microorganisms instead of the abbreviations or common names appearing in the result data). ‘Salmonella’ appears every year, while ‘Listeria monocytogenes’ appears eight times and ‘Escherichia coli’ appears five times in a decade. These three kinds of microorganisms are common pathogenic bacteria, and they are also the focus of biosafety research, as they have caused severe biosafety accidents throughout history [17, 55, 56]. In addition, the emergence of some microorganisms in the data is closely related to international emergent biosafety events, such as the appearance of ‘Ebola virus’ in 2014 and 2015 and ‘Zika virus’ in 2016 [32]. In particular, ‘MERS-CoV’ appeared in 2020 in addition to 2014 [45]. This phenomenon is likely related to the outbreak of COVID-19, which was also caused by a type of coronavirus [50]. Some microorganisms that do not directly cause human diseases, such as ‘RHDV’, also appear [16]. In addition, some keywords indicate other aspects of biosafety issues, such as ‘cyanobacteria’, which produce cyanotoxin. This toxin can harm water quality and threaten human health without adequate controls [51]. MERS-CoV and 2019-nCoV are viruses that spread through the air and can cause respiratory diseases [15, 31].

To learn more regarding the research relevance of various microorganisms, we used the microorganisms obtained from the data as keywords to search PubMed, processed the data with LDAPR and then searched for other microorganism keywords in the results. Finally, we used the chord diagram in Figure 6 to show the results. We found that much microbial research involves other microorganisms. Thus, studying the correlation among various microorganisms is also an important part of biosafety research. The research results from one problem can often inspire other work.

We found that the co-occurrence of microorganism-related keywords is closely related to the similarity of the microorganisms’ characteristics. For example, *Listeria monocytogenes* and Salmonella are food-borne pathogens that cause diarrhea and other symptoms [60].

**Figure 4:**
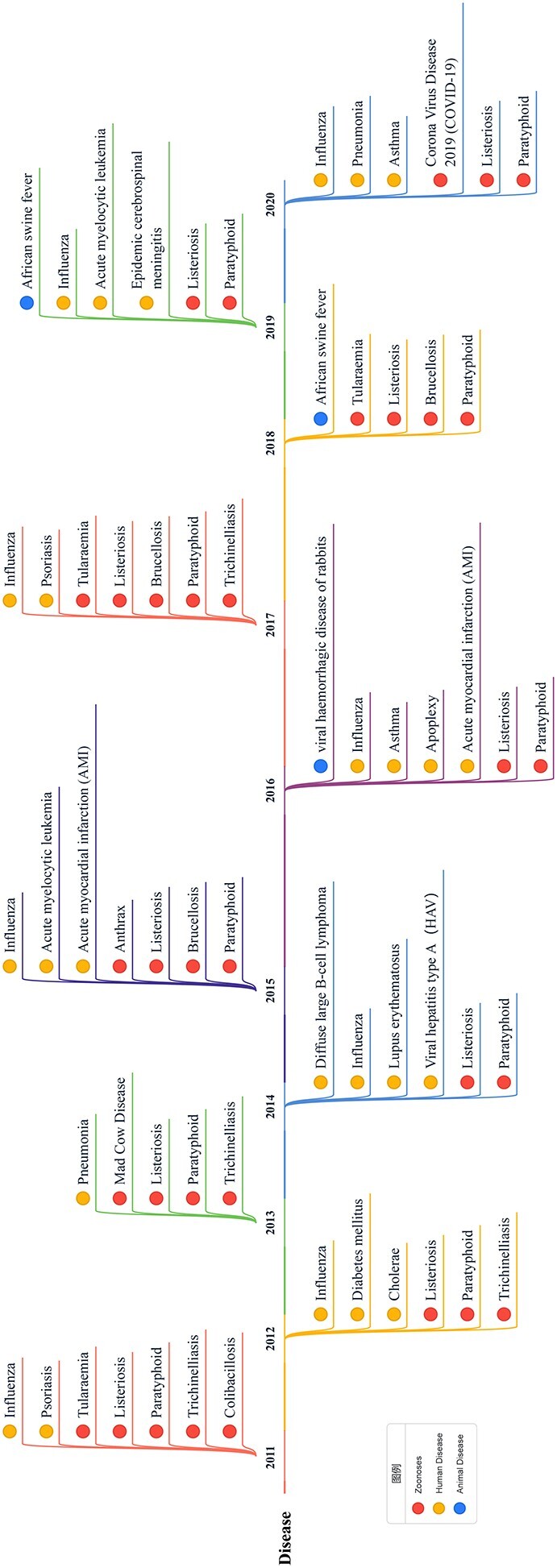
Disease-related keywords for each year from 2011 to 2020. We arrange the keywords in chronological order. Different colored dots represent different types of diseases, including zoonotic diseases (red), human diseases (yellow) and animal diseases (blue). Since no other types of diseases (such as plant diseases) are found in the results, we do not establish an ‘other’ category.

### Disease keywords

Biosecurity accidents are often accompanied by outbreaks of epidemic diseases, so we studied disease-related keywords and derived statistics on the results. Figure 4 shows the keywords in the disease field for each year. We divided diseases into three categories: ‘human disease’, ‘zoonoses’ and ‘animal diseases.’ Among them, items in the category ‘zoonoses’ appear most frequently, which means that most research is about zoonoses [39].

‘Listeriosis’ and ‘paratyphoid’ appear in all years. These are two common serious diseases caused by food safety problems. ‘Influenza’ appears eight times in 10 years. The prevention and control of influenza is thus an important topic in biosafety research [53].

By comparing the annual occurrence of microorganisms, it can be found that there is a strong correlation between diseases and microorganisms. For example, ‘meningococcus’ appears in the 2019 keywords, and ‘epidemic cerebrospinal meningitis’, which is caused by it, appears in the same year [38]. In addition, some diseases are not directly related to biosafety issues, but they are typical complications of certain diseases. For example, the items ‘apoplexy’ and ‘acute myocardial infarction (AMI)’ appearing in 2016 are typical complications of ‘ebola hemorrhagic fever’ [43]. To further explore the relationship between diseases and microorganisms, we calculated the co-occurrence of such pairs in the past 10 years; the results are shown in Figure 7 as a heat map.

In addition to diseases directly caused by pathogenic microorganisms, the frequency of the co-occurrence of certain diseases and other microorganisms is very high. For example, influenza and some infectious viruses often appear simultaneously. Viral infections usually cause influenza or are similar to influenza. The flu season is also when viruses spread most rapidly. On the disease side, the appearance of Listeria infection and paratyphoid are very similar. We can speculate that there is a close relationship between these two diseases. Trichinella and tularemia also have similar distributions, but the frequency of Trichinella infection is significantly higher than that of tularemia. Based on this, we conclude that tularemia is more likely to occur in groups at high risk for Trichinella infection, which has been confirmed by related studies [11]. On the microbial side, Salmonella is strongly associated with many diseases, which indicates that Salmonella infections tend to make patients more susceptible to other pathogens or that Salmonella-infected people are often exposed to an environment suitable for multiple pathogens [9].

**Figure 5:**
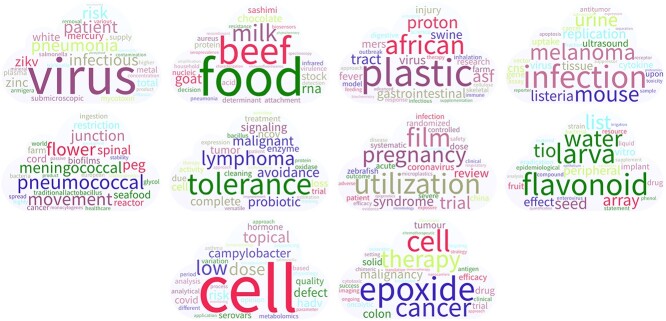
Word clouds for 2020. Each word cloud represents a topic, and the size of the word represents the weight within the topic.

**Figure 6:**
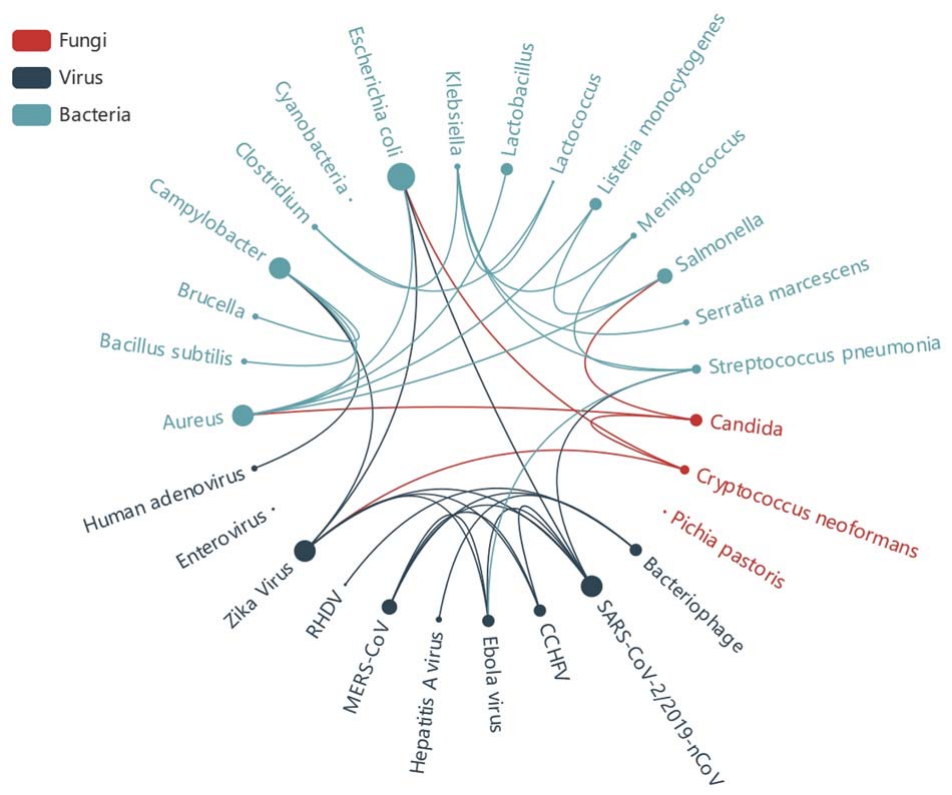
Co-occurrence of microorganisms. The lines between different microorganisms represent co-occurrence.

**Figure 7:**
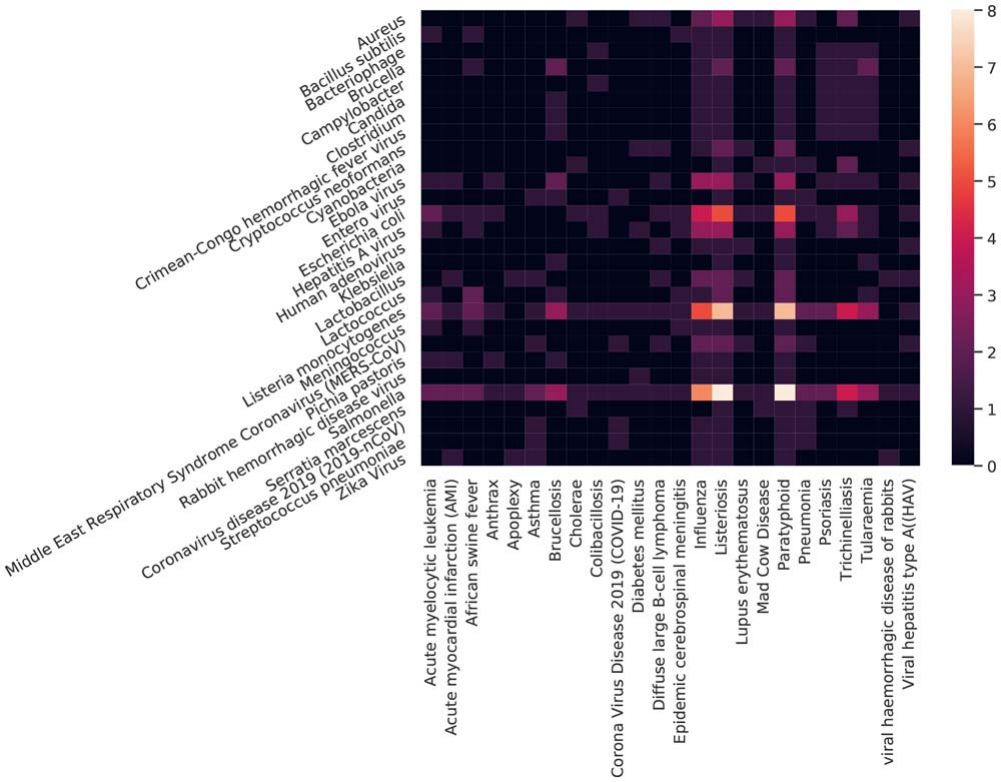
Co-occurrence of microorganisms and diseases. The lighter the color is, the more co-occurrences there are.

**Figure 8:**
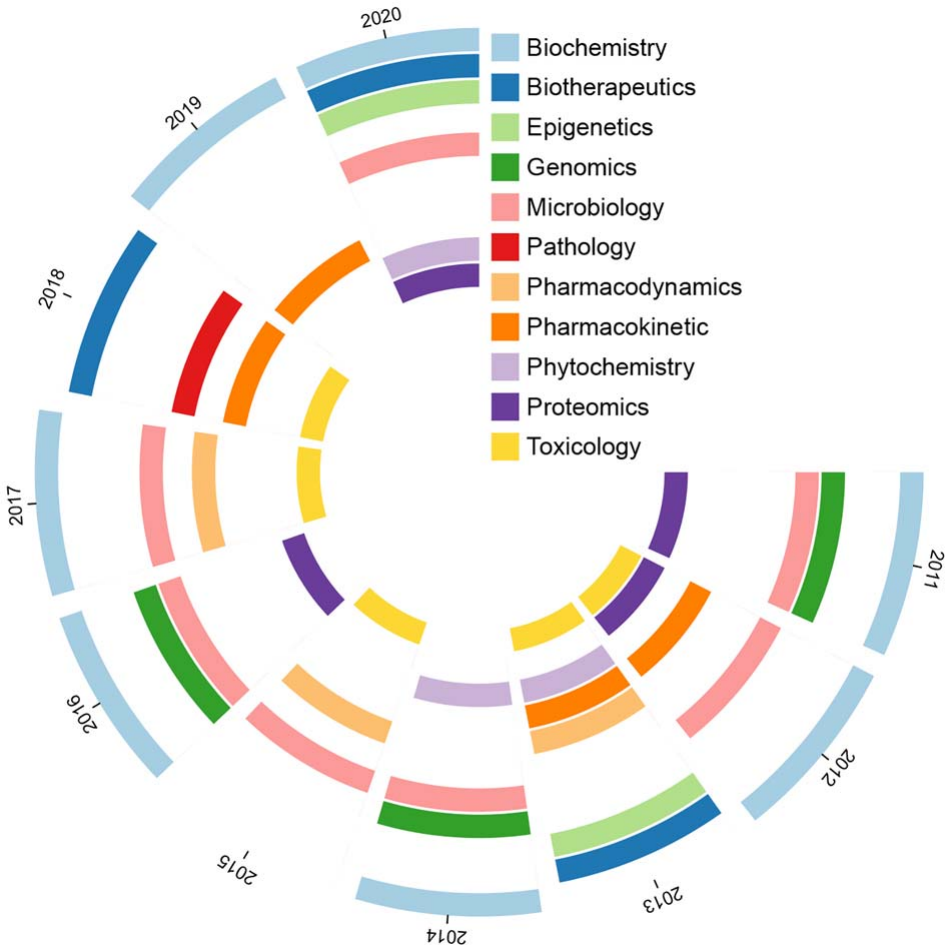
Disciplines involved in biosafety research in the decade studied. Different angles represent different years.

### Disciplinary keywords

It is widely acknowledged that biosafety research involves a variety of disciplines. From the results, we select each year’s disciplinary keywords for analysis; these are displayed in Figure 8.

We found publications from 2013 and 2020 corresponding to six disciplines, with the results in each year involving a variety of disciplines. Among them, publications from 2019 cover only two disciplines. ‘Biochemistry’ is associated with publications from 7 years (except 2013, 2015 and 2018), ‘microbiology’ with publications from 7 years (except 2013, 2018 and 2019) and ‘pathology’ with publications in 2018 only.

### Regional keywords

**Figure 9:**
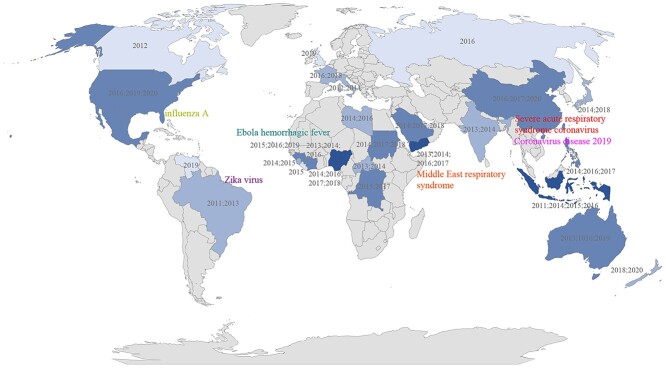
Hot regions in biosafety research. The shade of the color represents the keyword weight, and the years denote the time of keyword appearance.

Biosafety issues and related studies have strong regional characteristics. In Figure 9, we select and mark region-related keywords from the results on a world map and use different shades to represent the sums of the weights of regional keywords over the years to mark hotspots for biosafety research. Congo appears five times, while Australia, Indonesia and Saudi Arabia appear four times each. From the typical biosafety incidents marked in Figure 9, we can observe a strong correlation between mentions of these regions and significant biosafety incidents in the last decade [4]. For example, the regions involved in MERS are mainly in the Middle East, those involved in Ebola mainly in Central Africa, those involved in SARS mainly in East Asia and those involved in influenza A mainly in North America. The above regions all appear with high weights in the results.

### Research trends and challenges

**Figure 10:**
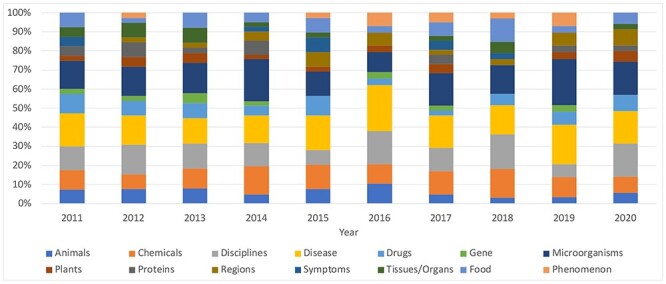
Keyword category ratio over the years. Items in the professional medical keyword categories (such as proteins and genes) are counted by comparison with the keywords in the MeSH database, while we manually count items in common keyword categories (such as animals and plants) ourselves.

Biosafety research is developing continuously, with many new research results appearing every year. To identify development trends, we collected keywords for each year separately. If we take 2017 as an example, the topic results are shown in the first subtable in Table 2. Unlike Table 1, which shows the central words only approximately, Table 2 shows the combined statistical results for all topics in each year. ‘Cell’, ‘gene’, ‘patient’ and ‘food’ are the central keywords. We visualize the proportions of keyword categories in the annual results in Figure 10.

From the results over the 10 years, we can observe the following: (1) ‘Cell’ always appears as a central keyword. It can be inferred that most of the research on biosafety is centered on cytology. (2) Words related to diseases, microorganisms, chemistry and disciplines appear in high proportions every year, indicating that these are long-term themes of biosafety research. (3) Biosafety is a discipline closely related to events. In addition to long-term research topics, there are some keywords related to public health emergencies, such as the keywords ‘coronavirus’, ‘pneumoniae’ and ‘vaccine’, which appear in 2020 and are closely related to the outbreak of COVID-19. (4) New research findings also bring about changes in research trends. For example, we found the term ‘pollen’ in the results for 2016 and 2017, indicating that research on pollen in the field of biosecurity may have delivered new findings. We then found evidence to support this speculation: the article ‘Pollen-mediated gene flow and seed exchange in small-scale Zambian maize farming, implications for biosafety assessment’ published in the journal *Nature* in October 2016 studied the impact of self-organized ecological factors (pollen flow) on biosafety [7].

### Topics over the decade

**Figure 11:**
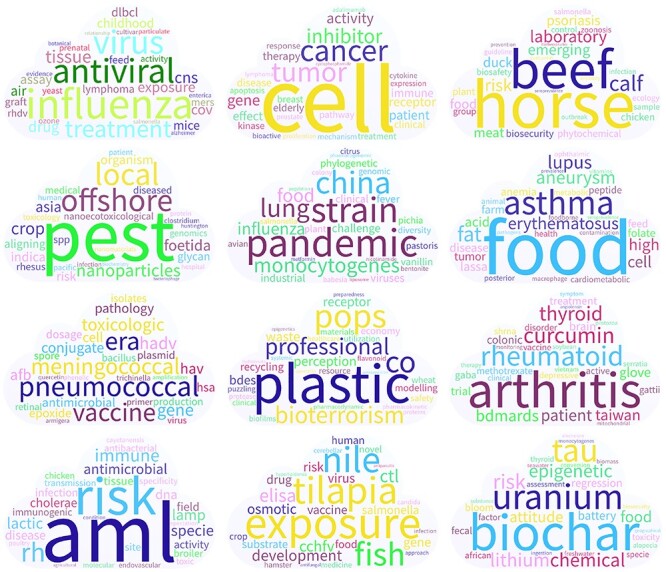
Decade word clouds.

To study the distribution of topics over the decade, we used LDAPR to process all the data and obtained 12 central topics. Then, we sorted these topics with their PageRank values. The results are shown in Figure 11.

Each word cloud represents a topic, and each topic corresponds to one or more of the topics in Figure 1. In this way, we can identify the latest trends and hotspots in biosafety-related research. For example, the central words of the first topic are ‘influenza’, ‘antiviral’, ‘virus’, RHDV and ‘MERS-CoV’, which are related to viruses and infections. It is well known that viruses and infections are primary topics in biosafety research. The second topic is probably related to cells and genetic diseases. With the development of modern medicine, diagnosis and treatment technology from the perspective of cells and genes has achieved remarkable results [12]. Biosafety research is increasingly turning to methods based on this series of modern medical technologies. The third topic is related mostly to animals. Food safety issues are related to the stability and development of society, and quarantines of meat products (such as pork, beef and mutton) are an

important topic in biosafety research [65]. With the rapid development of traditional meat production, processing industries and new meat products, related biosafety research is also facing new challenges. The fourth topic is associated with the environment. With the development of modern society, human activities have brought new challenges to environmental governance. Leakages of toxic and harmful biochemical agents and invasions of alien species are environmental issues related to biosafety [29, 55].

**Table 2:** Topics in years 2017, 2018, 2019 and 2020.

2017	2018	2019	2020
cell	cell	cell	cell
gene	risl	water	coronavirus
patient	human	virus	virus
food	food	drug	vaccine
farm	drug	food	mycotoxin
drug	trial	chronic	lung
mouse	exposure	farm	drug
infection	tumor	therapy	rna
bacteria	treatment	australia	salmonella
breast	cancer	aeruginosa	zikv
skin	metabolite	intravenous	mercury
isolate	safety	diabetic	zinc
antimicrobial	virus	protein	malignant
adipose	isolate	parasite	pneumoniae
biologics	chemical	lesion	clinical
cow	plant	infection	resistance
mechanism	africa	child	food
...	...	...	...

### Research on COVID-19

To validate the ability of our model to reflect emergency biosafety incidents, we performed an analysis of COVID-19-related research publications.

**Figure 12:**
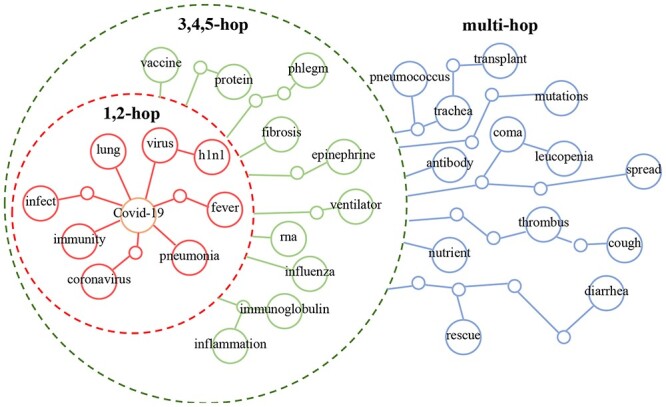
Results aligned with the COVID-19 knowledge graph. For intuitive interpretation, we group the results into 1,2-hop, 3,4,5-hop and multihop, respectively.

We align the results with the COVID-19 knowledge graph from [28] and show some of the content related to COVID-19 in Figure 12. It can be found that the hit words in our model are concentrated mainly around COVID-19. From the above results, we can observe the following: (1) Words directly related to COVID-19, such as ‘coronavirus’, ‘virus’, ‘pneumonia’ and ‘lung’, appear with higher weights, which indicates that our model can accurately reflect hotspot events. Based on this feature, we can discover more keywords strongly related to COVID-19 to support future research. (2) Weakly related words such as ‘infection’, ‘spread’ and ‘RNA’ also appear in the results with lower weights, which indicates the rationality of our model in weight distribution. Therefore, the weights of keywords can be used as references of their importance to assist with decision-making in COVID-19 research. (3) It is worth mentioning that indirectly related words such as ‘MERS’, ‘SARS’, ‘vaccine’ and ‘influenza’ have also been successfully mined, which shows the comprehensiveness of our model for information extraction. Therefore, the COVID-19 keyword output by the model does not simply reflect explicitly related information but can also cover implicitly related information.

## Method

As shown in Figure 2, our model accepts preprocessed paper abstracts crawled from PubMed by BioPython (https://biopython.org/) as an input. First, we use LDA to obtain preliminary topic results [5], train a word embedding model from the corpus with the Word2Vec language model [35, 41] and then process the topic results to construct a graph. Second, the topic-level weighted PageRank algorithm is used to rank these topics and remove noisy information [40]. Third, we use the AP clustering algorithm to obtain the clustering centers of the filtered results. Fourth, the keywords of each center are separately constructed as a graph, and then the word-level weighted PageRank algorithm is used to rerank the keyword results. Finally, we use tools to organize and visualize the results to identify development trends and research hotspots in biosafety research. We introduce the algorithms and overall framework of the model in the next sections.

### Latent Dirichlet allocation

LDA, a well-known unsupervised probability model for text topic extraction based on the Bayesian model, was proposed by Blei et al. in 2003 [6]. Its main purpose is to extract the hidden topics of each document from large-scale complex text information and use certain words to describe each topic to extract the key information of the text content [24, 68].

LDA divides the text content into three levels, namely, documents, topics and words, and expresses each document as a distribution of topics:

(1) \begin{align*}& P(w\mid d) = P(w \mid t) \times P(t \mid d), \end{align*}

where $w$, $t$ and $d$ represent words, topics and documents, respectively. This process can be further expressed as

(2) \begin{align*} &p(w_m,z_m,\theta_m, \Phi \mid \alpha, \beta ) = \nonumber\\ &\Pi _{n=1}^{N_m} p(\Phi \mid \beta)p(\theta_m \mid \alpha) p(z_{m,n}\mid \theta_{m})p(w_{m,n}\mid\Phi, z_{m,n}), \end{align*}

where $N_m$ represents the length of document $m$ and $z_{m,n}$ is the topic generated by document $m$.

To obtain the final topic results, we chose the Gibbs sampling method based on Markov chain Monte Carlo (MCMC) to estimate the LDA model [52].

### PageRank algorithm

PageRank is a webpage ranking algorithm proposed by Page et al. in 1998 [48]. It uses hyperlinks between pages as the main basis to measure the importance of pages by iteratively updating their PageRank values. That is, PageRank treats the hyperlinks between pages as votes to evaluate the importance of web pages and rank them by the number of votes. In our method, we assign a separate weight $w_{i,j}$ to each hyperlink [27, 71]. The update process among $K$ nodes can be expressed as follows:

(3) \begin{align*} PR_i^{l+1} &= \frac{1-\alpha}{\left \| K \right \|}+\alpha\left(\sum_{j\in N_i}\frac{w_{i,j}\cdot PR_j^l}{\left \| L_i \right \|}\right) \end{align*}

(4) \begin{align*} PR_i^0 &= \frac{1}{\left \| K \right \|}, \end{align*}

where $PR_i^{l}$ is the value of node $i$ with $N_i$ neighbors after $l$ updates. Here, $\alpha $ is the damping factor, and $L_i$ is the number of hyperlinks from page $i$.

In general, the PageRank algorithm uses more practical matrix operations to achieve the same effect. It can be expressed as

(5) \begin{align*} \begin{bmatrix} PR_1^{l+1}\\ PR_2^{l+1}\\ \vdots \\ PR_i^{l+1}\\ \end{bmatrix} = \begin{bmatrix} \frac{1-\alpha}{\left \| K \right \|}\\ \frac{1-\alpha}{\left \| K \right \|}\\ \vdots \\ \frac{1-\alpha}{\left \| K \right \|}\\ \end{bmatrix} + \alpha\begin{bmatrix} l_{1, } \ \ l_{1,2}\ \ \cdots\ \ l_{1,i}\\ l_{2,1} \ \ l_{2,2} \ \ \cdots \ \ l_{2,i}\\ \vdots \ \ \ \ \vdots \ \ \ \ \ddots \ \ \ \ \vdots \\ l_{3,1} \ \ l_{3,2} \ \ \cdots \ \ l_{i,i} \end{bmatrix} \cdot \begin{bmatrix} PR_1^{l}\\ PR_2^{l}\\ \vdots \\ PR_i^{l} \end{bmatrix} \end{align*}

(6) \begin{align*} l_{i,j} = \left\{\begin{array}{@{}ll} 0, & if \ w_{i,j} \ does \ not \ exist \\ w_{i,j}, & otherwise \end{array}.\right. \end{align*}

Through the above formula, the PageRank value $PR_{i,j}$ of each node is continuously updated until convergence, which is the final ranking result.

### Affinity propagation clustering

AP is a graph-clustering algorithm proposed in 2007 [20]. It selects ‘exemplars’ through ‘message passing’ among nodes. Unlike traditional clustering methods, it can use existing data as ‘exemplars’ to represent the corresponding category. AP clustering has achieved good results in many fields [22, 37, 73]. We tried many clustering algorithms here. AP is the best choice based on both the principle and the experimental results. Moreover, AP does not need to specify the number of centers and is insensitive to the initial value, making it very suitable for text data with high dimensions and uncertainty.

There are four main parameter matrices in the AP algorithm: similarity $s(i,k)$, preference $p(k)$, responsibility $r(i,k)$ and availability $a(i,k)$, where $s(i,k)$ is the similarity between node $i$ and node $k$, represented here using the Euclidean distance, and where $p(k)$ is the similarity value when $i=k$. In the initial state, all responsibility and availability values are initialized to 0, and they are updated according to the following equations:

(7) \begin{align*}& r_{t+1}(i,k) = \left\{\begin{array}{@{}ll} S(i,k)-max_{j\neq k}\{a_t(i,j)+r_t(i,j)\},& i\neq k\\ S(i,k)-max_{j\neq k}\{S(i,j)\}, & i= k \end{array}\right. \end{align*}

(8) \begin{align*} &a_{t+1}(i,k) = \nonumber\\ &\left\{\begin{array}{@{}ll} min\{0, \ r_{t+1}(k+k)+\sum_{j\neq i,k}max\{r_{t+1}(j,k), \ 0\}\},& i\neq k\\ \sum_{j\neq k} max\{r_{t+1}(j,k), \ 0\}, & i= k \end{array}\right. \end{align*}

(9) \begin{align*}& r_{t+1}(i,k) = \lambda \cdot r_t(i,k) + (1-\lambda)\cdot r_{t+1}(i,k) \end{align*}

(10) \begin{align*}& a_{t+1}(i,k) = \lambda \cdot a_t(i,k) + (1-\lambda)\cdot a_{t+1}(i,k), \end{align*}

where $\lambda $ is the damping factor.

After the iterative update, we calculate the sum of each pair of $s(i,k)$ and $p(i,k)$. For node $i$, if the $k$ value that maximizes $a(i,k)+r(i,k)$ is $k^{\prime}$, then if $i=k^{\prime}$, point $i$ is the cluster center; otherwise, node $i$ belongs to center $k^{\prime}$.

### Overall framework

We obtained the abstracts of 66 758 related papers published during the past 10 years from the biomedical database PubMed. The keywords included ‘biosafety’, ‘bio-safety’, ‘biosecurity’, ‘bio-security’, ‘biological safety’ and ‘biohazard’.

The statistical results show that with the development of biotechnology and increased social attention to biosafety-related fields, related research is also increasing. Figure 14 shows the number of related papers published over the years, revealing an increasing year-on-year trend.

**Figure 13:**
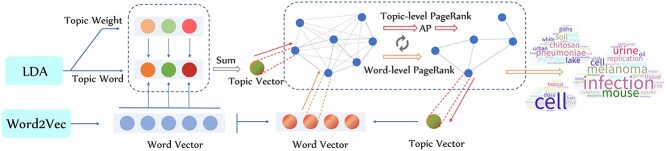
Main part of the LDAPR model. The data flow is from left to right in the direction of the arrow and is processed by each module. The dots in the figure represent words (topic words), scalars (word weights) or vectors (word vectors and topic vectors). Although we describe the two levels of the PageRank algorithm as the same process for the sake of simplicity, the contents of network composition in the two stages are notably different.

**Figure 14:**
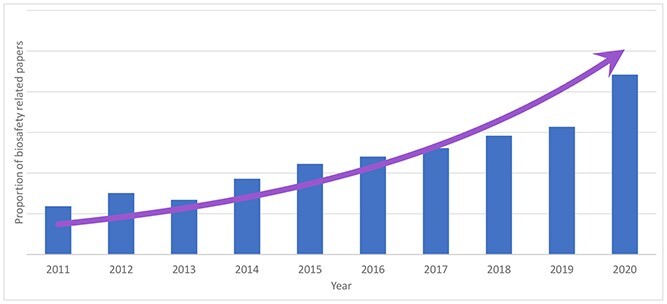
Number and trend of biosafety-related research papers published from 2011 to 2020. The number of papers published each year shows a steadily increasing trend year on year, with the growth rate in 2020 in comparison to the level in 2011 being approximately 204.86%.

The original data obtained from PubMed are natural language text and contain a great deal of noisy information. To achieve better results for subsequent work, we preprocess the data with natural language processing tools (such as term frequency inverse document frequency [TF-IDF], dictionary building and bag-of-words [BOW] models).

Next, we introduce the main part of the model. The structure and flow of this part are shown in Figure 13. We obtain $T$ topics from the data by the LDA model, with $K$ words in each one, and we use the corpus to train a Word2Vec language model to obtain the word embedding vector of each word. Finally, the word embedding vectors of all topic words are weighted using the weights assigned by the LDA model and summed according to the dimensions of the topic, expressed by the formula:

(11) \begin{align*}& \nu^{topic}_{t} = \sum_{k=1}^{K}{w_{t,k}\nu^{word}_{t,k}}, \end{align*}

where $\nu ^{topic}_{t}$ represents the vector of the $t$-th topic and $t=\{1,2,...,T\}$. $K$ represents the number of keywords selected under each topic, and $w_{t,k}$ and $\nu ^{word}_{t,k}$ represent the weight and word vector of the $k$-th keyword of the $t$-th topic, respectively. Thus far, we have obtained the representation of each topic.

We connect all topics in pairs and use the cosine similarity between their topic vectors as the weights of the edges to construct an undirected weighted graph. The formula is as follows:

(12) \begin{align*}& C_{i,j} = \frac{\sum_{d=1}^{D}(T_i^d\times T_j^d)}{\sqrt{\sum_{d=1}^{D}(T_i^d)^2} \cdot \sqrt{\sum_{d=1}^{D}(T_j^d)^2}}, \end{align*}

where $C_{h,t}$ represents the weight of the edge between topic vector nodes $V_h$ and $V_t$ and $h=\{1,2,...,T\},t=\{1,2,...,T\},h\neq t$. Then, we construct the topic graph $G_t$ and delete the edges with lower weights according to the threshold $K_t$. Therefore, the value of nodes $O^t$ and the weight of edges $E^t$ in the initial state are:

(13) \begin{align*}& O_i^T = \frac{1}{\left \| T \right \|}; \end{align*}

(14) \begin{align*}& E^t_{i,j} = \left\{\begin{array}{@{}ll@{}} 0, &if \ i = j \ or \ C_{i,j}< K_t \\ C_{i,j},& otherwise \end{array},\right. \end{align*}

where $T$ is the number of topics. Next, we use topic-level weighted PageRank to iteratively update this network and remove low-ranking topics. This step aims to remove noisy topics as much as possible before further processing.

Then, we use the AP algorithm to cluster the remaining topics and select the central topics of each category as the representatives. Subsequently, we redecouple each central topic as a set of topic words and construct word networks with weighted edges from Equation 12 according to the cosine similarity between word vectors. Similar to the topic graph construction method, we use a threshold $K_w$ to filter out edges with low weights. Then, word-level weighted PageRank is used to rerank the topic words to remove noise and obtain the final results. Unlike the previous topic-level PageRank, here, we take words as nodes to build a separate graph for each topic. Finally, we use word clouds, time series graphs, radar graphs, chord graphs, heat maps, world maps, etc., to visualize the results from multiple angles and select representative results for visualization purposes. More details can be viewed on our homepage(https://www.keaml.cn/Biosafety/) .

### Hyperparameter settings

**Figure 15:**
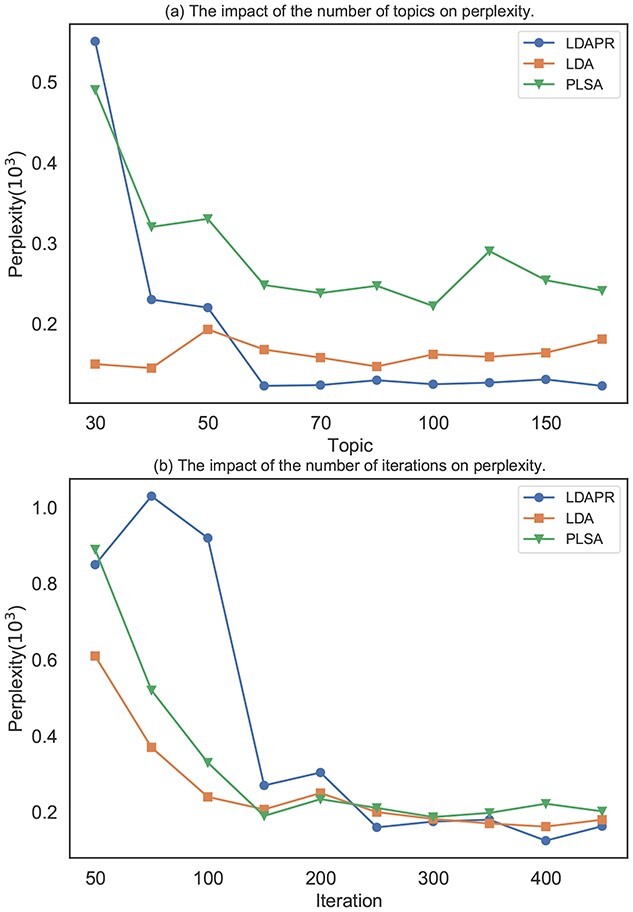
Sensitivity to the number of topics **(**A) and iterations **(**B). (Comparison with LDA [6] and probabilistic latent semantic analysis [PLSA] [26]. )

We tune the hyperparameters of the model based on perplexity [72],

(15) \begin{align*} perplexity(D)=exp\left(-\frac{\sum log p(w)}{\sum_{M}^{d=1}N_d} \right) \nonumber\\ p(w) = p(z|d)* p(w|z), \end{align*}

and we show the impact of two key factors, the numbers of topics and iterations, on perplexity in Figure 15. For topic models, perplexity is a commonly used metric. Intuitively, perplexity represents how ambiguous the topic results are, so we choose the hyperparameters that give the model a lower perplexity. In our model, we set $\alpha =0.15$ and $\beta =0.01$. For the AP model, the damping factor is set to 0.95 after we test many alternatives. For the PageRank models, the topic-level damping factor is $\alpha _t=0.85$, the word-level damping factor is $\alpha _w=0.45$ and by observing the convergence of the PageRank value, we set the model to iterate 50 times for both levels.

### Performance comparison

To demonstrate the effectiveness of our method, we selected **Random**, **LDA** and **PLSA** as the baseline models to compare with LDAPR. The results of perplexity($\times 10^3$) of Random, LDA, PLSA and LDAPR are 17.909, 0.222, 0.162 and 0.125. And the *P*-value of LDAPR with Random, LDA and PLSA are $0.1059\times 10^{-5}$, $0.4694\times 10^{-4}$ and 0.0014. We find that LDAPR shows significant and consistent improvement over the other methods.

To verify the effectiveness of each component of LDAPR, we conduct ablation experiments on the language models (**BERT**[33], **BioBERT**[36] and **pretrained FastText**[8]) and clustering algorithms (**K-means**[34, 67]; we choose the point closest to the center as the center point) . The results of perplexity ($\times 10^3$) of -K-means, -Pretrained FastText, -BERT, -BioBERT and LDAPR are 0.386, 0.242, 0.324, 0.207 and 0.125. Although BERT is a great language model, it does not perform well due to the constraints of the task and corpus. And compared with other methods, our model achieved the best performance. The experimental results demonstrate that the selection of each component is reasonable and effective.

### More information

To discover more information, we expand the numbers of topics and words. Specifically, we use the selected keywords to filter the results and calculate the weight $W$ of each keyword as

(16) \begin{align*}& W_{k} = \frac{exp(W^{\prime}_{k}/\tau)}{\sum_{n=1}^{K}exp(W^{\prime}_{n}/\tau)}; \end{align*}

(17) \begin{align*}& W^{\prime}_{k}=\sum_{1}^{T}W_{t} W_{t,k}, \end{align*}

where $T$ is the number of topics, $W_{t}$ is the weight of topic $t$, $W_{t,k}$ is the weight of keyword $k$ in topic $t$ and $\tau $ is the temperature parameter.

**Figure 16:**
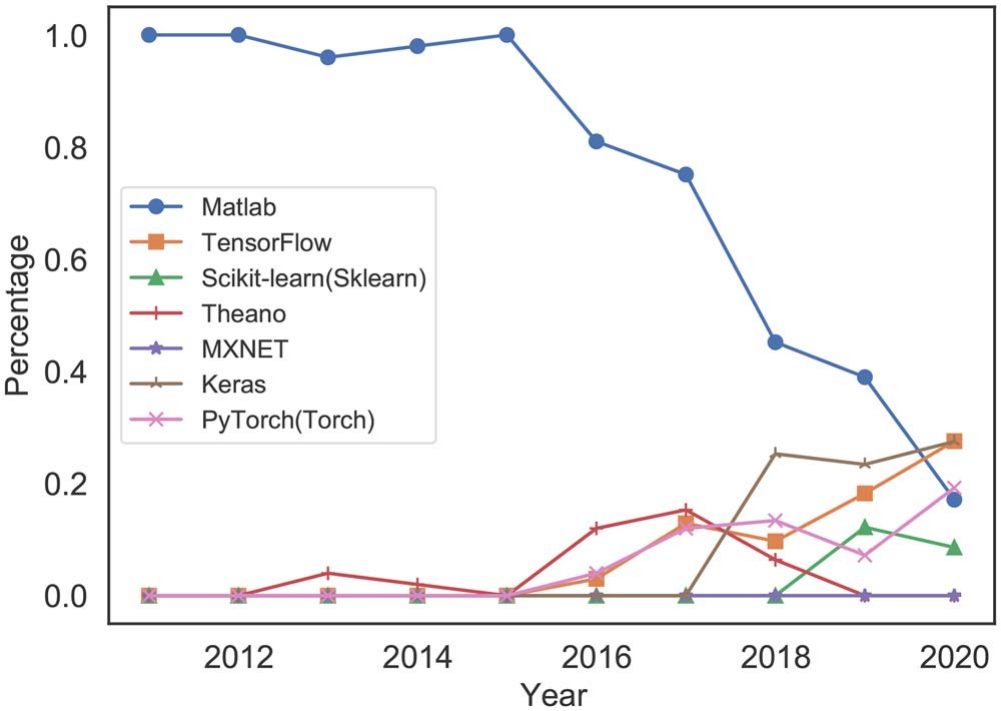
‘Exemplars’ of trend knowledge patterns captured by the models for bioinformatics frameworks or tools. Here, ‘Matlab’ stands for the toolboxes contained in Matlab, and we refer to these toolboxes and other deep learning libraries as ‘research tools’.

Taking bioinformatics frameworks and tools as an example, we use relevant keywords to search, and the results are presented in Figure 16. Through trend analysis, we can clearly capture trends in biosafety research tools. In the early stage, Matlab occupied the core position among research tools. During this period, researchers used mainly the data analysis methods and modeling toolboxes in Matlab for data mining. Since 2015, with the rapid rise of deep learning, researchers have begun to pay attention to the use of deep learning frameworks such as TensorFlow for pattern recognition. Google promoted TensorFlow during this period. Although deep learning frameworks generally show an upward trend, Theano was no longer popular after 2016, mainly because the Montreal Institute for Learning Algorithms (MILA) stopped supporting it at that time.

## Conclusion

In this paper, we proposed a novel LDAPR framework based on LDA, AP and the hierarchical PageRank algorithm to summarize and analyze trends in biosafety research over the past decade. We processed 66 758 papers in related fields and visualized and fully analyzed the results. Studies in related fields support our results, which proves the comprehensiveness and accuracy of our method. The research objects of this experiment covered many areas. Among them, we focused on the microorganisms, diseases and disciplines associated with biosafety research. We discovered many implicit connections among these categories and extracted valuable information that is expected to be useful for research. We believe that the LDAPR model can play a guiding role in trend analysis for bodies of biosafety literature and can shed light on the future directions of biosafety research. For example, it can be seen from the results that research on microorganisms, especially infectious disease viruses, has gradually become an important research hotspot. Genes and vaccines are increasingly becoming the topics of greatest concern in biosafety research.

**Key Points**

- With the outbreak and spread of COVID-19, biosafety has become a global hot topic, and a large number of related studies have emerged.
- Compared with other research domains, biosafety research covers more fields and more disciplines. Therefore, it is difficult to systematically organize and summarize the enormous number of related research papers.
- We developed a novel topic model: LDAPR. We utilized this model to process biosafety-related papers in the past 10 years and to discover and analyze trends and hotspots.
- We discovered a large number of implicit relationships in the data and demonstrated their authenticity and accuracy via relevant studies.
- The proposed model can also be applied to many research fields and can provide valuable information for future research.

## Data Availability

All data relevant to the study are included in the article or uploaded as supplementary information.

## Author Biographies

**Renchu Guan** is a professor in the College of Computer Science and Technology at Jilin University, China. His research interests include machine learning, bioinformatics, and knowledge engineering.

**Haoyu Pang** is an undergraduate student in the College of Computer Science and Technology at Jilin University, China. His research interests include data mining and recommender systems.

**Yanchun Liang** is a professor in the College of Computer Science and Technology at Jilin University, Changchun, China. He is also a professor at Zhuhai Laboratory of Key Laboratory of Symbol Computation and Knowledge Engineering of Ministry of Education, Zhuhai College of Science and Technology, Zhuhai, China. His research interests include computational intelligence, machine learning methods, text mining, and bioinformatics.

**Zhongjun Shao** is a professor in the School of Public Health at Air Force Medical University, China. His research activity concerns epidemiology and preventive medicine.

**Xin Gao** is a Professor in Computer Science, Associate Director of Computational Bioscience Research Center, and Deputy Director of Smart Health Initiative at King Abdullah University of Science and Technology (KAUST), Saudi Arabia. His research interests focus on bioinformatics and machine learning.

**Dong Xu** is Curators' Distinguished Professor in the Department of Electrical Engineering and Computer Science at the University of Missouri. His research is in computational biology and bioinformatics, including machine-learning application in bioinformatics, protein structure prediction, post-translational modification prediction, and high-throughput biological data analyses.

**Xiaoyue Feng** is an associate professor in the College of Computer Science and Technology at Jilin University, China. Her research activity concerns machine learning in biomedical applications.

## References

[1] Ali M , Abbasi BH, Ahmad N, et al. Over-the-counter medicines in Pakistan: misuse and overuse. The Lancet 2020;395(10218):116. 10.1016/S0140-6736(19)32999-X 31929013

[2] Alimonti J , Leung A, Jones S, et al. Evaluation of transmission risks associated with in vivo replication of several high containment pathogens in a biosafety level 4 laboratory. Sci Rep 2014;4(1):1–7. 10.1038/srep05824 PMC5376055 25059478

[3] Andersson DI . Persistence of antibiotic resistant bacteria. Curr Opin Microbiol 2003;6(5):452–6. 14572536 10.1016/j.mib.2003.09.001

[4] Bauch CT , Oraby T. Assessing the pandemic potential of mers-cov. The Lancet 2013;382(9893):662–4. 10.1016/S0140-6736(13)61504-4 PMC7137078 23831143

[5] Blei DM , Lafferty JD. A correlated topic model of science. The Annals of Applied Statistics 2007;1(1):17–35.

[6] Blei DM , Ng AY, Jordan MI. Latent dirichlet allocation. *The*. Journal of Machine Learning Research 2003;3:993–1022.

[7] Bøhn T , Aheto DW, Mwangala FS, et al. Pollen-mediated gene flow and seed exchange in small-scale zambian maize farming, implications for biosafety assessment. Sci Rep 2016;6(1):1–12. 27694819 10.1038/srep34483 PMC5046111

[8] Bojanowski P , Grave E, Joulin A, et al. Enriching word vectors with subword information. Transactions of the association for computational linguistics 2017;5:135–46.

[9] Bula-Rudas FJ , Rathore MH, Maraqa NF. Salmonella infections in childhood. Adv Pediatr 2015;62(1):29–58. 26205108 10.1016/j.yapd.2015.04.005

[10] Callaway E . Biosafety concerns for labs in the developing world. Nature 2012;485(7399):425. 22622543 10.1038/485425a

[11] Carvalho C , De Carvalho IL, Zé-Zé L, et al. Tularaemia: a challenging zoonosis. Comp Immunol Microbiol Infect Dis 2014;37(2):85–96. 24480622 10.1016/j.cimid.2014.01.002 PMC7124367

[12] Catena R , Santos E, Orive G, et al. Improvement of the monitoring and biosafety of encapsulated cells using the sfgnestgl triple reporter system. J Control Release 2010;146(1):93–8. 20580914 10.1016/j.jconrel.2010.05.018

[13] Celis C , Scurrah M, Cowgill S, et al. Environmental biosafety and transgenic potato in a Centre of diversity for this crop. Nature 2004;432(7014):222–5. 15538370 10.1038/nature03048

[14] Christian MD . Biowarfare and bioterrorism. Crit Care Clin 2013;29(3):717–56. 23830660 10.1016/j.ccc.2013.03.015 PMC7127345

[15] Cornish NE , Anderson NL, Arambula DG, et al. Clinical laboratory biosafety gaps: lessons learned from past outbreaks reveal a path to a safer future. Clin Microbiol Rev 2021;34(3):e00126–18. 10.1128/CMR.00126-18 PMC8262806 34105993

[16] Dalton KP , Nicieza I, Balseiro A, et al. Variant rabbit hemorrhagic disease virus in young rabbits, Spain. Emerg Infect Dis 2012;18(12):2009. 23171812 10.3201/eid1812.120341 PMC3557890

[17] Denamur E , Clermont O, Bonacorsi S, et al. The population genetics of pathogenic escherichia coli. Nat Rev Microbiol 2021;19(1):37–54. 32826992 10.1038/s41579-020-0416-x

[18] Do DT , Le NQK. Using extreme gradient boosting to identify origin of replication in saccharomyces cerevisiae via hybrid features. Genomics 2020;112(3):2445–51. 31987913 10.1016/j.ygeno.2020.01.017

[19] Eggers B , Mackenzie R. The Cartagena protocol on biosafety. Journal of International Economic Law 2000;3(3):525–43.

[20] Frey BJ , Dueck D. Clustering by passing messages between data points. Science 2007;315(5814):972–6. 17218491 10.1126/science.1136800

[21] Gao GF . For a better world: biosafety strategies to protect global health, 2019. 10.1016/j.bsheal.2019.03.001 PMC7147920 32572394

[22] Guan R , Shi X, Marchese M, et al. Text clustering with seeds affinity propagation. IEEE Transactions on Knowledge and Data Engineering 2011;23(4):627–37.

[23] Hanson R , Sulkin S, Buescher E, et al. Arbovirus infections of laboratory workers: extent of problem emphasizes the need for more effective measures to reduce hazards. Science 1967;158(3806):1283–6. 6058003 10.1126/science.158.3806.1283

[24] L. Hennig . Topic-based multi-document summarization with probabilistic latent semantic analysis. In Proceedings of the International Conference RANLP-2009, pages 144–9, 2009.

[25] Hodgson J . Biosafety rules get thumbs up. Nat Biotechnol 2000;18(3):253–3. 10.1038/73669 10700125

[26] Hofmann T . Probabilistic latent semantic analysis. In Proceedings of the Fifteenth conference on Uncertainty in artificial intelligence, pp. 289–96, 1999.

[27] Hu J , Chen H, Heidari AA, et al. Orthogonal learning covariance matrix for defects of grey wolf optimizer: insights, balance, diversity, and feature selection. Knowledge-Based Systems 2021;213:106684.

[28] L. Huang , H. Guan, Y. Liang, R. Guan, and X. Feng. Covid-19 knowledge graph for drug and vaccine development. In 2021 IEEE International Conference on Bioinformatics and Biomedicine (BIBM), pages 328–33. IEEE, 2021.

[29] Hulme PE . Invasion pathways at a crossroad: policy and research challenges for managing alien species introductions. J Appl Ecol 2015;52(6):1418–24.

[30] Hung TNK , Le NQK, Le NH, et al. An ai-based prediction model for drug-drug interactions in osteoporosis and paget’s diseases from smiles. Molecular Informatics 2022. 10.1002/minf.202100264 34989149

[31] Jiang C , Yao X, Zhao Y, et al. Comparative review of respiratory diseases caused by coronaviruses and influenza a viruses during epidemic season. Microbes Infect 2020;22(6–7):236–44. 32405236 10.1016/j.micinf.2020.05.005 PMC7217786

[32] Katzelnick LC , Narvaez C, Arguello S, et al. Zika virus infection enhances future risk of severe dengue disease. Science 2020;369(6507):1123–8. 32855339 10.1126/science.abb6143 PMC8274975

[33] J. D. M.-W. C. Kenton and L. K. Toutanova. Bert: Pre-training of deep bidirectional transformers for language understanding. In Proceedings of NAACL-HLT, pages 4171–86, 2019.

[34] Krishna K , Murty MN. Genetic k-means algorithm. IEEE Trans Syst Man Cybern B Cybern 1999;29(3):433–9. 18252317 10.1109/3477.764879

[35] Q. Le and T. Mikolov. Distributed representations of sentences and documents. In International Conference on Machine Learning, pages 1188–96. PMLR, 2014.

[36] Lee J , Yoon W, Kim S, et al. Biobert: a pre-trained biomedical language representation model for biomedical text mining. Bioinformatics 2020;36(4):1234–40. 31501885 10.1093/bioinformatics/btz682 PMC7703786

[37] Leone M , Weigt M. Clustering by soft-constraint affinity propagation: applications to gene-expression data. Bioinformatics 2007;23(20):2708–15. 17895277 10.1093/bioinformatics/btm414

[38] Masignani V , Pizza M, Moxon ER. The development of a vaccine against meningococcus b using reverse vaccinology. Front Immunol 2019;10:751. 31040844 10.3389/fimmu.2019.00751 PMC6477034

[39] Meslin F . Global aspects of emerging and potential zoonoses: a who perspective. Emerg Infect Dis 1997;3(2):223. 9204308 10.3201/eid0302.970220 PMC2627609

[40] R. Mihalcea and P. Tarau. Textrank: Bringing order into text. In Proceedings of the 2004 conference on empirical methods in natural language processing, pages 404–11, 2004.

[41] Mikolov T , Sutskever I, Chen K, Corrado GS, Dean J. Distributed representations of words and phrases and their compositionality. Adv Neural Inf Process Syst 2013;26.

[42] Morens DM , Folkers GK, Fauci AS. The challenge of emerging and re-emerging infectious diseases. Nature 2004;430(6996):242–9. 15241422 10.1038/nature02759 PMC7094993

[43] Mulangu S , Dodd LE, Davey RT, Jr, et al. A randomized, controlled trial of ebola virus disease therapeutics. N Engl J Med 2019;381(24):2293–303. 31774950 10.1056/NEJMoa1910993 PMC10680050

[44] Neri S . Genetic stability of mesenchymal stromal cells for regenerative medicine applications: a fundamental biosafety aspect. Int J Mol Sci 2019;20(10):2406. 10.3390/ijms20102406 PMC6566307 31096604

[45] Oboho IK , Tomczyk SM, Al-Asmari AM, et al. 2014 mers-cov outbreak in Jeddah-a link to health care facilities. N Engl J Med 2015;372(9):846–54. 25714162 10.1056/NEJMoa1408636 PMC5710730

[46] Olaimat AN , Shahbaz HM, Fatima N, et al. Food safety during and after the era of covid-19 pandemic. Front Microbiol 2020;11:1854. 32849446 10.3389/fmicb.2020.01854 PMC7417330

[47] Pack RL . The complex move of plum island research center to Manhattan, Kansas and potential policy considerations. Drake J Agric L 2018;23:511.

[48] Page L , Brin S, Motwani R, et al. The pagerank citation ranking: Bringing order to the web. Technical report, Stanford InfoLab, 1999.

[49] Pattnaik S , Swain K, Lin Z. Graphene and graphene-based nanocomposites: biomedical applications and biosafety. J Mater Chem B 2016;4(48):7813–31. 32263772 10.1039/c6tb02086k

[50] Petrosillo N , Viceconte G, Ergonul O, et al. Covid-19, sars and mers: are they closely related? Clin Microbiol Infect 2020;26(6):729–34. 32234451 10.1016/j.cmi.2020.03.026 PMC7176926

[51] Plaas HE , Paerl HW. Toxic cyanobacteria: a growing threat to water and air quality. Environ Sci Technol 2020;55(1):44–64. 33334098 10.1021/acs.est.0c06653

[52] I. Porteous , D. Newman, A. Ihler, A. Asuncion, P. Smyth, and M. Welling. Fast collapsed gibbs sampling for latent dirichlet allocation. In Proceedings of the 14th ACM SIGKDD International Conference on Knowledge Discovery and Data Mining, pages 569–77, 2008.

[53] Potter CW . A history of influenza. J Appl Microbiol 2001;91(4):572–9. 11576290 10.1046/j.1365-2672.2001.01492.x

[54] Pyšek P , Hulme PE, Simberloff D, et al. Scientists’ warning on invasive alien species. Biol Rev 2020;95(6):1511–34. 32588508 10.1111/brv.12627 PMC7687187

[55] Rai PK , Singh J. Invasive alien plant species: their impact on environment, ecosystem services and human health. Ecol Indic 2020;111:106020. 32372880 10.1016/j.ecolind.2019.106020 PMC7194640

[56] Rogers AW , Tsolis RM, Bäumler AJ. Salmonella versus the microbiome. Microbiol Mol Biol Rev 2021;85(1):e00027–19. 33361269 10.1128/MMBR.00027-19 PMC8549850

[57] Salata C , Calistri A, Alvisi G, et al. Ebola virus entry: from molecular characterization to drug discovery. Viruses 2019;11(3):274. 10.3390/v11030274 PMC6466262 30893774

[58] Singhal T . A review of coronavirus disease-2019 (covid-19). The Indian Journal of Pediatrics 2020;87(4):281–6. 32166607 10.1007/s12098-020-03263-6 PMC7090728

[59] Strauss A , White A, Boots M. Invading with biological weapons: the importance of disease-mediated invasions. Functional Ecology 2012;26(6):1249–61.

[60] Tauxe RV . Emerging foodborne pathogens. Int J Food Microbiol 2002;78(1–2):31–41. 12222636 10.1016/s0168-1605(02)00232-5

[61] N. S.-O. I. A. H. V. I. Team . Emergence of a novel swine-origin influenza a (h1n1) virus in humans. N Engl J Med 2009;360(25):2605–15. 19423869 10.1056/NEJMoa0903810

[62] Tomori O , Kolawole MO. Ebola virus disease: current vaccine solutions. Curr Opin Immunol 2021;71:27–33. 33873076 10.1016/j.coi.2021.03.008

[63] Trevan T . Biological research: rethink biosafety. Nature 2015;527(7577):155. 26560283 10.1038/527155a

[64] Van Duin D , Paterson DL. Multidrug-resistant bacteria in the community: trends and lessons learned. Infectious Disease Clinics 2016;30(2):377–90. 27208764 10.1016/j.idc.2016.02.004 PMC5314345

[65] Wang D , Liang Y, Xu D, et al. A content-based recommender system for computer science publications. Knowledge-Based Systems 2018;157:1–9.

[66] Wang F , Zhang W. Synthetic biology: recent progress, biosafety and biosecurity concerns, and possible solutions. Journal of Biosafety and Biosecurity 2019;1(1):22–30.

[67] Wang M , Chen H. Chaotic multi-swarm whale optimizer boosted support vector machine for medical diagnosis. Appl Soft Comput 2020;88:105946.

[68] X. Wei and W. B. Croft. Lda-based document models for ad-hoc retrieval. In Proceedings of the 29th Annual International ACM SIGIR Conference on Research and Development in Information Retrieval, pages 178–85, 2006.

[69] Weiss S , Yitzhaki S, Shapira SC. Lessons to be learned from recent biosafety incidents in the United States. The Israel Medical Association Journal: IMAJ 2015;17(5):269–73. 26137650

[70] Whittaker RJ , Fernández-Palacios JM, Matthews TJ, et al. Island biogeography: taking the long view of nature’s laboratories. Science 2017;357(6354). 10.1126/science.aam8326 28860356

[71] W. Xing and A. Ghorbani. Weighted pagerank algorithm. In Proceedings. Second Annual Conference on Communication Networks and Services Research, 2004*.*, pages 305–14. IEEE, 2004.

[72] X. Yan , J. Guo, Y. Lan, and X. Cheng. A biterm topic model for short texts. In Proceedings of the 22nd international conference on World Wide Web, pages 1445–56, 2013.

[73] Yang C , Bruzzone L, Sun F, et al. A fuzzy-statistics-based affinity propagation technique for clustering in multispectral images. IEEE Transactions on Geoscience and Remote Sensing 2010;48(6):2647–59.

[74] Zhou D , Song H, Wang J, et al. Biosafety and biosecurity. Journal of Biosafety and Biosecurity 2019;1(1):15–8. 32501430 10.1016/j.jobb.2019.01.001 PMC7148603

